# Ayka, a Novel *Curtobacterium* Bacteriophage, Provides Protection against Soybean Bacterial Wilt and Tan Spot

**DOI:** 10.3390/ijms231810913

**Published:** 2022-09-18

**Authors:** Rashit I. Tarakanov, Anna A. Lukianova, Peter V. Evseev, Roksana I. Pilik, Anna D. Tokmakova, Eugene E. Kulikov, Stepan V. Toshchakov, Alexander N. Ignatov, Fevzi S.-U. Dzhalilov, Konstantin A. Miroshnikov

**Affiliations:** 1 Department of Plant Protection, Russian State Agrarian University—Moscow Timiryazev Agricultural Academy, Timiryazevskaya Str. 49, 127434 Moscow, Russia; 2 Shemyakin-Ovchinnikov Institute of Bioorganic Chemistry, Russian Academy of Sciences, Miklukho-Maklaya Str., 16/10, 117997 Moscow, Russia; 3 Agrobiotechnology Department, Agrarian and Technological Institute, RUDN University, Miklukho-Maklaya Str., 6, 117198 Moscow, Russia; 4 School of Biological and Medical Physics, Moscow Institute of Physics and Technology, National Research University, Institutskiy Per, 9, Dolgoprudny, 141701 Moscow, Russia; 5 Research Center of Biotechnology, Winogradsky Institute of Microbiology, Russian Academy of Sciences, Prosp. 60-Letia Oktyabrya, 7-2, 117312 Moscow, Russia; 6 Center for Genome Research, National Research Center “Kurchatov Institute”, Kurchatov Sq., 1, 123098 Moscow, Russia

**Keywords:** *Curtobacterium*, soybean, wilt, bacteriophage, genomics, phage control

## Abstract

Diseases caused by the Gram-positive bacterium *Curtobacterium* *flaccumfaciens* pv. *flaccumfaciens* (Cff) inflict substantial economic losses in soybean cultivation. Use of specific bacterial viruses (bacteriophages) for treatment of seeds and plants to prevent the development of bacterial infections is a promising approach for bioprotection in agriculture. Phage control has been successfully tested for a number of staple crops. However, this approach has never been applied to treat bacterial diseases of legumes caused by Cff, and no specific bacteriophages have been known to date. This paper presents detailed characteristics of the first lytic bacteriophage infecting this pathogen. Phage Ayka, related to φ29-like (*Salasmaviridae*) viruses, but representing a new subfamily, was shown to control the development of bacterial wilt and tan spot in vitro and in greenhouse plants.

## 1. Introduction

In recent years, legumes (soybeans, beans, peas, etc.) have become an important export crop for the Russian Federation. Legumes are affected by phytopathogenic fungi, oomycetes, bacteria and viruses. Bacterial pathogens pose a particular danger due to the multifactorial nature of transmission of infection (by seeds, insects, nematodes, rainwater, etc.), the lack of diagnostic tools and the lack of effective methods for combatting bacterial diseases of legumes. The situation is complicated by poor knowledge of both the specific and intraspecific composition of the populations of phytopathogenic bacteria affecting leguminous crops and related species in the crop rotation. Monitoring studies of the species composition of soybean pathogens in Russia have shown that a noticeable fraction of bacterial diseases is presented as brown blight, tan spot or wilting caused by *Curtobacterium flaccumfaciens* (Hedges, 1922) Collins and Jones 1984 [1]. This species of Gram-positive bacteria is known to be a phytopathogen but has not been considered among the main causative agents of bacterial diseases of legumes, and the data on this pathogen are sparse [1]. Thorough investigations in recent decades have revealed a pathovar, *C. flaccumfaciens* pv. *flaccumfaciens* (further in the text Cff) that primarily infects beans and soybeans [2,3]. Cff attacks the vascular system of the plant, causing leaf spots, burns, wilting and the death of seedlings and adult plants. Infected seedlings are retarded in growth, their leaves fall off, shoots die off and the main stem breaks. Non-deadly infection of this pathogen reduces yield [4] and seed quality [5]. Cff has been listed by the European and Mediterranean Plant Protection Organisation (EPPO) on the A2 List of Quarantine Subjects (https://www.eppo.int, accessed on 28 October 2021) (PM 1/002 (28)) (PM 7/102 (1)). The pathogen is included in the lists of quarantine objects of group A1 in Egypt, Morocco, South Africa, Tunisia, Argentina, Chile, Paraguay, Uruguay, Bahrain, China, Israel, Jordan and Turkey (RPPO/EU APPPC A1, CAHFSA, COSAVE (A2), EPPO (A2), EU (A1 Quarantine pest), IAPSC (A1 list).

Infected seeds are the main source of infection [6]. Thus, Cff has a potentially high risk of spreading in Russia, primarily due to the rapid development of soybean cultivation. Currently, the conventional way to reduce losses from disease is prevention. Preventive measures include the use of certified seeds [2,3,6] and resistant cultivars [4,7,8,9]. Due to their low efficiency, the complexity of certification and regulation of use, and economic considerations, the use of chemical bactericides that can protect plants from Cff is limited [4,10]. Biological protection against bacterial infections is considered to be the most promising and innovative method. Special attention is paid to the use of bacteriophages (bacterial viruses), which are natural regulators of microbial communities. Biocontrol using natural phages infecting specific species and strains of bacteria is an extremely attractive way to combat phytopathogens due to high specificity and biosecurity. This method also has several limitations associated with the excessive specificity of bacteriophages and the low resistance of viral particles to extreme external conditions [11,12,13]. To overcome a number of information gaps and ambiguities, a systematic study is required, which will provide a scientifically substantiated and effective use of bacteriophage preparations for protecting leguminous plants from Cff.

## 2. Results

### 2.1. Bacterial Strains

From preliminary work [14], the authors have identified a number of field strains attributed as *C. flaccumfaciens* pv *flaccumfaciens* by MALDI-TOF [15], multilocus sequence typing (MLST) and PCR assays using genus-specific [16] and species-specific [17] primers. Bacterial strains were isolated from infected seeds of soybean cvs. Dauria and Kasatka in the Stavropol, Ryazan, Orel and Amur regions of the Russian Federation. In spite of these regions being geographically distant, the strains were notably similar. However, the Cff collection strains, especially ones affecting other crops, showed a high diversity in terms of their genomic and phytopathologic properties.

Cff strain C089, denoted F30-1 in [14], was chosen as a propagation host for phage Ayka. The ANI calculations indicated that the genome of C089 (GenBank accession number JANUGT000000000) is very close to the type strain of *Curtobacterium flaccumfaciens* pv. *flaccumfaciens* CFBP3418 = LMG3645 (99.97% of average nucleotide identity).

### 2.2. Isolation of Phages Specific to Curtobacterium Flaccumfaciens pv. Flaccumfaciens

Circulating strains C086-C091 (Supplementary Table S1) were used as components of the enrichment culture for phage isolation. A total of 22 attempts at phage isolation were made using field soil from the Belgorod, Kursk, Moscow, Saratov and Penza regions and Karelia Republic, river water from the Kurgan region and Tatarstan and Bashkortostan republics, sewage water from the Moscow region, and infected seed material. The phages infecting Cff strains (n = 10) were found in soil samples only. Based on plaque appearance, particle morphology revealed by EM and host range, isolated phages were almost identical. Therefore, a phage named Ayka, isolated from a field near Srednyaya Elyuzan village in Penza region (geographical coordinates of sampling spot 52°59′40′′ N and 45°56′44′′ E), was used for further detailed study.

### 2.3. Biological Properties of Curtobacterium Phage Ayka

#### 2.3.1. Morphology

**Figure 1:**
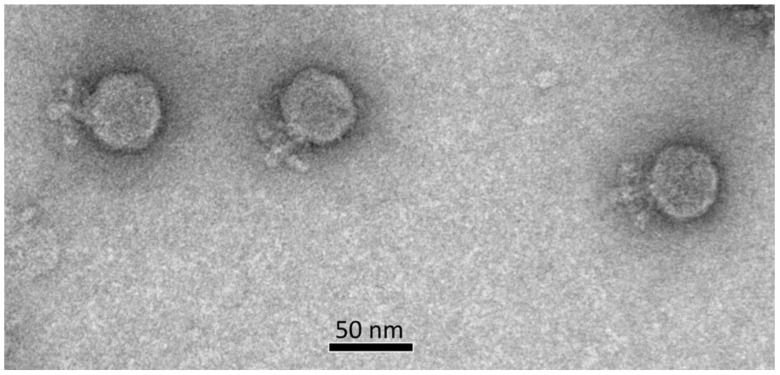
Electron microscopy image of phage Ayka. The scale bar is 50 nm.

As revealed by transmission electron microscopy (Figure 1), the virions of phage Ayka feature a podoviral morphology with an isometric capsid with a diameter of ~48 nm measured face to face. The length of the short tail is about 23 nm. The tail is surrounded by appendages of ~14 nm in length. Therefore, the universal nomenclature considering phage morphology [18] suggests the vB_CffP_Ayka name. The phage formed similar small plaques (Ø1–2 mm) with smooth borders (Supplementary Figure S1) on the lawn of the bacterial host C089 grown on 0.7% upper YD agar.

#### 2.3.2. Phage Production and Stability

Phage Ayka adsorbed to Cff host strain C089 cells almost completely (87%) in 13 min (Supplementary Figure S2A) at 26 °C, and lysed bacteria in 80 min, forming 102 ± 4 progeny particles per infected bacterial cell (Supplementary Figure S2B). UV (280–315 nm) irradiation decreased phage viability proportionally to the period of treatment (Supplementary Figure S3A). Complete UV destruction occurred within 5 h. Phage survival at temperatures from 4 to 70 °C was evaluated. The viability of phage Ayka dropped significantly at temperatures above 60 °C (Supplementary Figure S3B). Specifically, a phage suspension with a concentration of 10^7^ PFU/mL lost 50% viability at 60 °C within 1 h. The optimal long storage temperature for the phages was about 4 °C. The phage was stable in SM buffer with pH 5–10 at 26 °C for 1 h (Supplementary Figure S3C), but lost viability rapidly at pH 3–4 and pH 11–12. Supplementary Figure S3D shows that the optimal MOI of Ayka was 0.01.

The host range of the Ayka phage is comparatively narrow. Of the 74 strains tested, the phage is active only against nine (Supplementary Table S1). It is noteworthy that the phage lysed all six strains isolated in 2021 from soybean cultivars (C086–C091), even though the samples for bacterial isolation were delivered from geographically distant regions. In addition, the strain lysed one of the three strains isolated in the same year from peas (C101). Additionally, the phage lyses two more collection strains, namely VKM Ac-2055 (C017) and 400DL (C130).

All susceptible bacterial strains yielded a positive signal in the PCR test [17], but several strains that are also positive according to this criterion, including type Cff strain (C001), remained resistant to phage Ayka. This suggests that the phage is specific for some strain groups within the pathovar. The phage was avirulent to all tested non-*Curtobacterium* strains, including *Bradyrhizobium japonicum*, applied for soybean seed treatment. Thus, phage Ayka can be considered safe for conventional nitrogen-fixating microorganisms.

### 2.4. Genomic Properties of Phage Ayka

#### 2.4.1. General Genomic Features

*Curtobacterium* phage Ayka (GenBank accession number ON381767) is a double-stranded DNA virus with a genome size of 18,400 base pairs. The G+C-content of the genome is 52.6% and is noticeably lower than the typical G+C-content of *Curtobacterium flaccumfaciens* strains (about 71%). There are 22 open reading frames (ORFs) predicted in the Ayka genome, and are all located on the forward strand. Putative functions were assigned to 16 genes and six genes were annotated as hypothetical protein coding sequences (Supplementary Table S2). The genome contains no tRNA genes.

**Figure 2:**
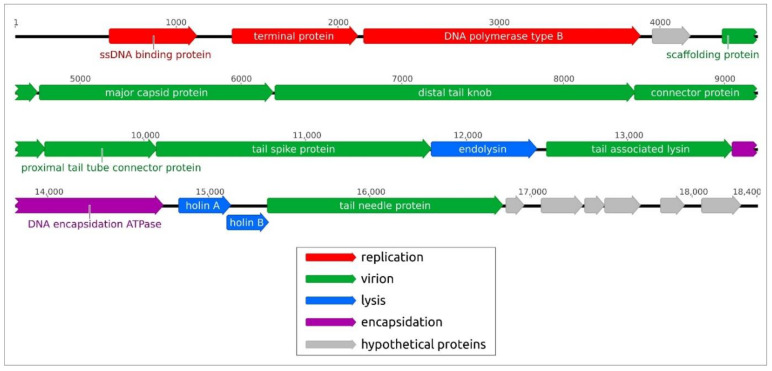
Genetic map of *Curtobacterium* phage Ayka. Arrows indicate the direction of transcription. The genes encoding hypothetical proteins are coloured grey.

The genome (Figure 2) contains the block of DNA-processing genes including the gene encoding the terminal protein. The presence of this gene indicates the protein-primed replication mechanism [19] that is typical for a number of small-tailed phages related to φ29 and other *Salasmaviridae* phages. The list of morphogenesis proteins includes a φ29-like major capsid protein and several other proteins that are similar to φ29 proteins, according to the results of HMM-HMM comparisons. Notably, gene16 was predicted to encode the tail needle protein, which is reminiscent of the structure of *Staphylococcus aureus* phage P68. The tail needle protein of phage P68 can participate in forming a pore in the bacterial membrane for delivering phage DNA into the bacterial cytoplasm [20]. The adsorption apparatus comprises tail spikes with predicted acetylglucosaminidase activity (gene product 10, gp10). The cassette of lysis proteins comprises endolysin and two holins, and the packaging apparatus includes a φ29-like terminase (DNA encapsidation ATPase, gp13) (Figure 2).

#### 2.4.2. Intergenomic Comparisons, Phylogeny and Taxonomy

To ascertain the phages related to phage Ayka, a BLAST search using the phage GenBank database and sequences of predicted proteins was conducted. The search revealed several dozen phages of Gram-positive bacteria and a *Rhizobium* phage (RHph_N3_8) which possessed some local homologies with phage Ayka. The major capsid protein, terminase, and DNA polymerase, and a number of other structural and lysis proteins of phage Ayka, were most similar to actinophages belonging to the genus *Anjalivirus*. The major capsid protein proposedly has the canonical HK97 fold.

**Figure 3:**
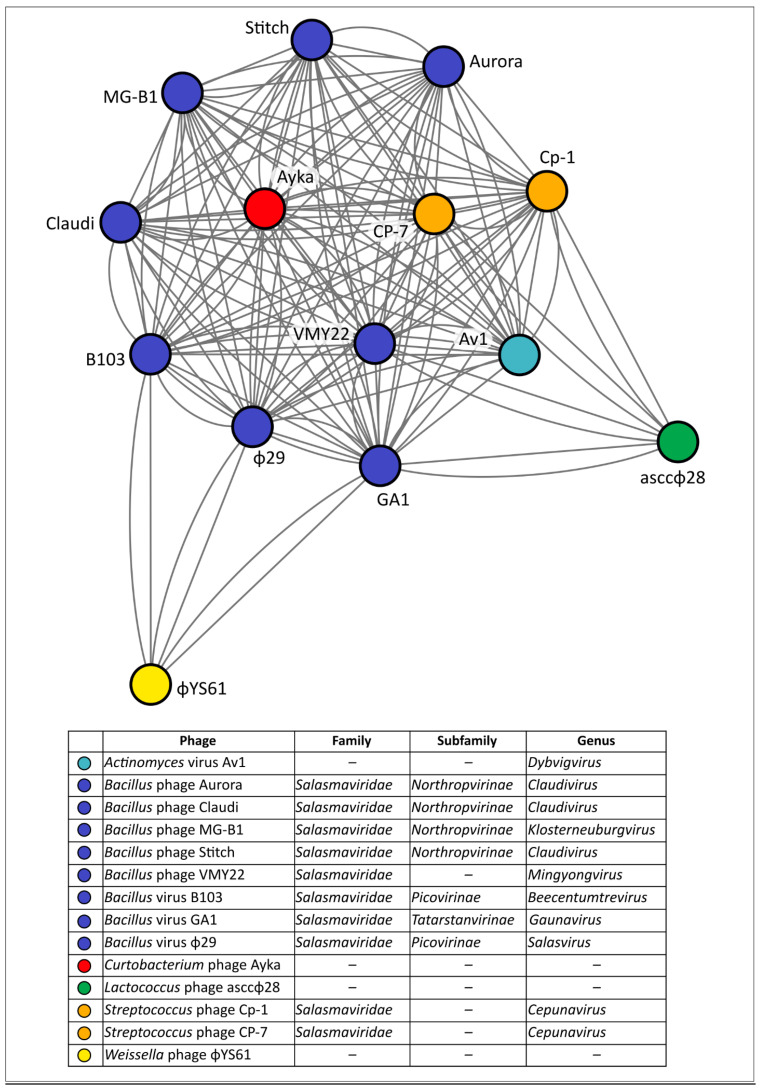
Gene-sharing network cluster containing *Curtobacterium* phage Ayka and related phages created using the vConTACT.2.0 and ProkaryoticViralRefSeq database v94 supplied with vConTACT.2.0.

An intergenomic distance comparison using the genomic sequences of phage Ayka and related phages, performed with the Virus Intergenomic Distance Calculator (VIRIDIC) [21], indicated a level intergenomic similarity compared with related phages of about 5% and less, which is much lower than the 70% genus threshold (Supplementary Figure S4). This does not enable assignment of the phage Ayka to any genus recognised by the International Committee on Taxonomy of Viruses (ICTV). However, a network analysis using the gene-sharing network obtained with the vConTACT.2.0 pipeline (Figure 3) indicated that the phage Ayka clusters with *Bacillus* phage φ29 and related phages, most of which are classified as members of the *Salasmaviridae* family. The phylogenetic tree created using GRAViTy also placed the phage Ayka and *Salasmaviridae* phages in sister groups (Supplementary Figure S5).

**Figure 4:**
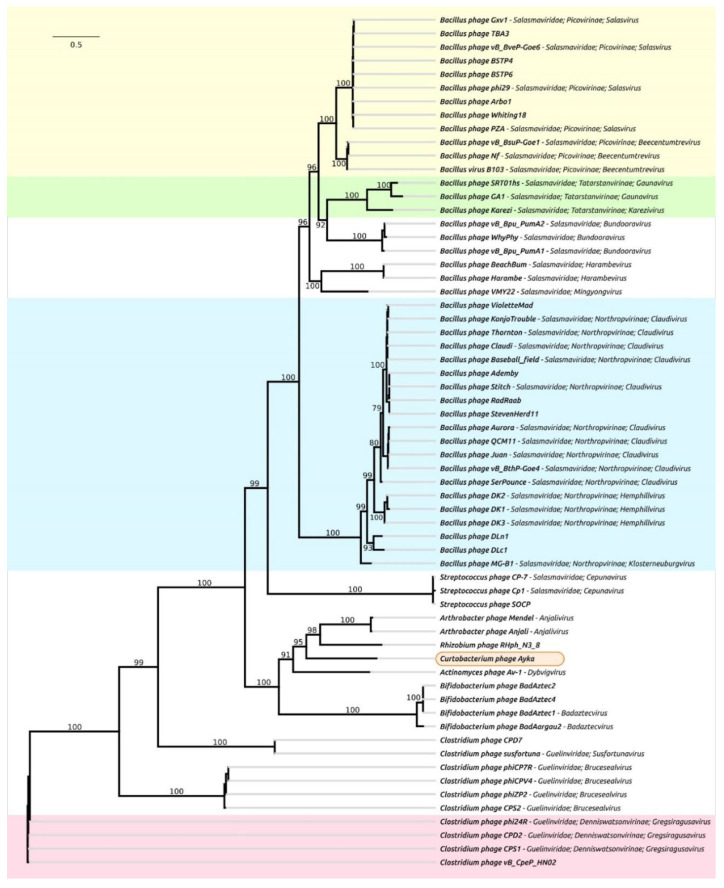
Best-scoring phylogenetic trees constructed with RAxML-NG based on the concatenated protein sequences of major capsid protein, terminase, DNA polymerase, connector protein and proximal tail tube connector protein. Bootstrap support values are shown above their branch as a percentage of 1000 replicates. The scale bar shows 0.5 estimated substitutions per site and the tree was unrooted. Clades of classified subfamilies are marked with different backgrounds.

A phylogenetic analysis using protein sequences of major capsid protein, terminase, DNA polymerase, connector protein and proximal tail tube connector protein indicated similar topologies of trees and a similar list of related phages (Supplementary Figures S6–S10). The phylogenetic tree constructed using the concatenated sequences of these five proteins is depicted in Figure 4. The tree places *Curtobacterium* phage Ayka into one clade with *Actinomyces* phage Av-1 of the *Dybvigvirus* genus, *Arthobacter* phages of the *Anjalivirus* genus and unclassified *Rhizobium* phage RHph_N3_8. The genetic distances between these three groups of phages and phage Ayka are of the same order as the genetic distances between the classified subfamilies of the *Salasmaviridae* family, which is also related to the Ayka phage, but more distantly (Figure 4).

**Figure 5:**
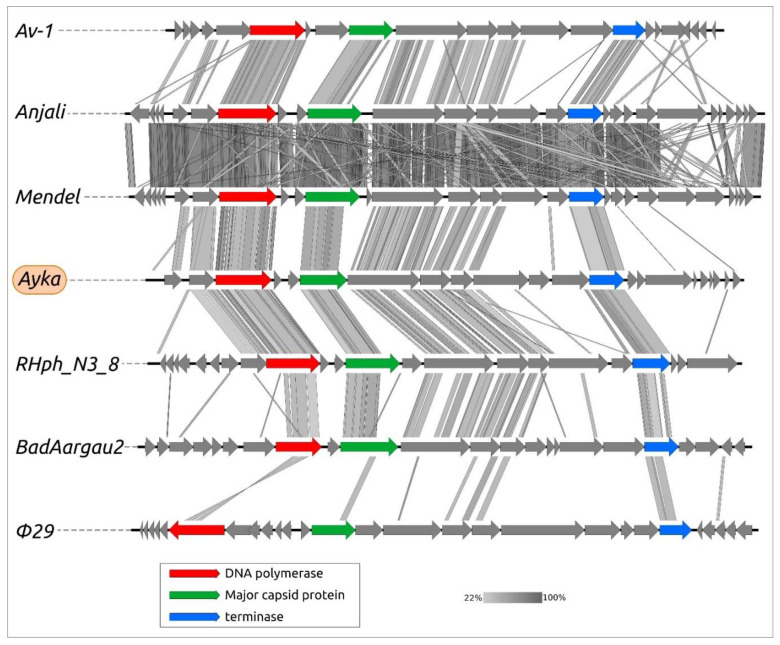
Genome sequence comparison among phage genomes exhibiting co-linearity detected by TBLASTX. Phage abbreviations are as follows: Av-1—*Actinomyces* phage Av-1, Anjali—*Arthobacter* phage Anjali, Mendel—*Arthobacter* phage Mendel, Ayka—*Curtobacterium* phage Ayka, RHph_N3_8—*Rhizobium* phage RHph_N3_8, BadAargau2—*Bifidobacterium* phage BadAargau2, φ29—*Bacillus* phage φ29. The percentage of sequence similarity is indicated by the intensity of the grey. Vertical boxes between the analysed sequences indicate areas with a similarity of at least 22%, shown on the scale below. The genes are coloured according to their function in the legend.

The results of the comparison of genomic sequences of phage Ayka and related phages (Figure 5) are consistent with the conclusions of phylogenetic analysis. The comparison demonstrates a similar genomic organisation of *Curtobacterium* phage Ayka and related phages, including *Actinomyces* phage Av-1 of the *Dybvigvirus* genus, *Arthobacter* phages of the *Anjalivirus* genus, *Rhizobium* phage RHph_N3_8 and *Bifidobacterium* phages of the *Badaztecvirus* genus. The comparison also indicates a higher level of conservation of structural proteins, terminase and DNA polymerase compared with other proteins, which makes it possible to reveal homologies between the above phages, on the one hand, and *Bacillus* φ29 phage, on the other.

### 2.5. Phage control of Curtobacterium Infection on Soybean

#### 2.5.1. Effect of Phage Treatment of Cff Leaf Infection

**Figure 6:**
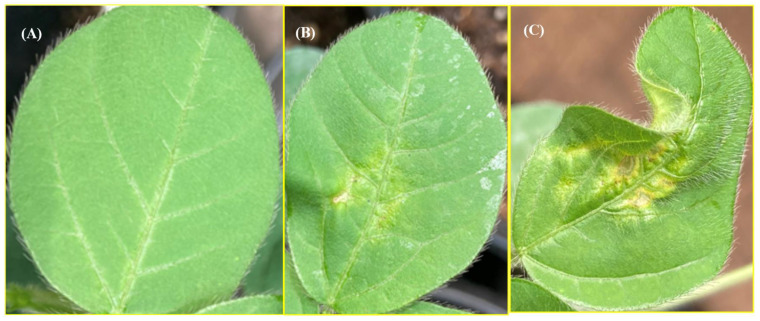
Symptoms of Cff on soybean leaves 12 days after infection of the leaves with an airbrush. (**A**) Control without inoculation (Cff infection); (**B**) treatment with phage Ayka; (**C**) positive control (water treatment of infected leaves). Panel B presents variant CFF+Phage (t = 1 h). Typical leaves from the samples are presented.

Cff-infected leaves of soybean were treated with the suspension of phage Ayka in triplicate. Disease development was assessed using LeafDoctor software, 12 days after the application of phage onto previously infected plants. It was also possible to visually detect the difference in the degree of damage to the soybean leaf. In particular, when treated with phage Ayka, the area of leaf damage was reduced (Figure 6B), compared with the positive control treated with water (Figure 6C). On a leaf without bacteria, symptoms did not appear (Figure 6A).

**Figure 7:**
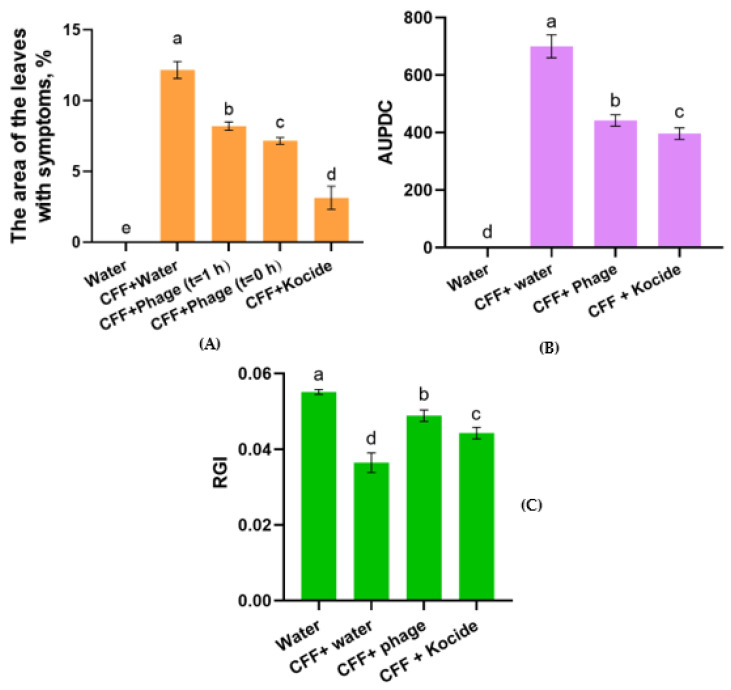
The effect of phage Ayka and standard bactericide Kocide on the area of lesions of the leaf surface (**A**), the Area Under Progress Disease Curve (AUPDC) of wilting (**B**) and the relative growth index (RGI) (**C**) of soybean in greenhouse conditions. Values in panels represent the respective mean of three independent trials; error bars represent the standard deviation. Values within columns marked by different letters indicate a significant difference, using Duncan’s criterion, *p* = 0.05.

As a result of phage application, progress of the disease was reduced by 32 and 41% (with application 1 h post-infection and simultaneously with the infection, respectively), compared with the positive control (Figure 7A). Inoculation of the leaves with an airbrush made it possible to achieve a persistent infection with typical Cff symptoms, manifested in the occurrence of chlorosis and necrosis of the leaves 12 days after treatment. No visible mechanical damage from the action of the airbrush was observed. It is worth noting that the smallest area of lesions on the leaf surface was observed after the application of a standard bactericide Kocide (3.14% of the leaf area), while phage control resulted in 8.2 and 7.2% damage. The use of the phage post-infection had less effect compared with the simultaneous application of bacteria and phage. This can be explained by the development of the pathogen in the intercellular space, where the phage cannot easily reach. The effective delivery of the phage requires the development of a phage formulation that is able to reach the affected site of the leaf.

#### 2.5.2. Effect of Phage Treatment of Cff Seed Infection

The method of seed infection by damaging and inoculating seeds in a vacuum in the presence of a suspension of bacteria allowed us to obtain symptoms typical of Cff in the form of wilting and stunting of soybean seedlings. The treatment of the soybean seeds previously infected with Cff using phage Ayka demonstrated a significant reduction in infection frequency and rate of disease development in the seedlings. The control treatment using water resulted in the fast occurrence of the symptoms with an average AUPDC (Area Under Progress Disease Curve) of up to 700 (Figure 7B). The application of phage Ayka to the seeds infected with Cff yielded a lower frequency of infected seedlings and disease development rate. The biological efficiency of phage control was 36.8% AUPDC compared with the control. AUPDC after the use of Kocide was lower than that of phage treatment (396 units in triplicate for bactericide and 442 units for phage Ayka).

Considering that the pathogen produces phytotoxic glycopeptides [22], the positive control showed a reduction in RGI (Relative Growth Index), while the use of phage and Kocide treatment showed higher values (Figure 7C). The reduction of RGI in the case of Kocide treatment can be explained by the phytotoxic effect of copper hydroxide on seedlings [23]. Thus, the copper-based bactericide had a greater effect on the pathogen, but also inhibited the relative index of the soybean growth.

## 3. Discussion

The use of bacteriophages is an alternative strategy to control bacterial diseases in integrated systems of plant protection [13]. Recent research has aimed to isolate and comprehensively characterise new lytic phages, and to assess their practical potential for plant protection [12]. Some bacterial pathogens have been shown to have been successfully treated with phage compositions (reviewed in [13]), while the application of phage control to other phytopathogenic bacteria is hindered by difficulties with phage delivery to the vascular system of plants, the instability of phages, or insufficient knowledge of the diversity of the strains of pathogens and respective phages. For example, phages infective to *Curtobacterium flaccumfaciens* pv. *flaccumfaciens* and to *Curtobacterium* sp. in general were not known at the time of this study.

Cff attracted the attention of phytopathologists after a number of serious epiphytoties of staple legumes. The pathogen survives poorly in soil, remaining viable for 77 to 154 days in laboratory conditions and for less than 90 days in natural clay [24]. Contaminated seeds of beans and soybeans are considered to be a major source of infection, especially in regions where the disease has not been reported previously [3,25].

No reliable protocols for preventive antibacterial treatment of bean and soybean seeds have been reported. Treatment with copper compounds and antibiotics results in an unstable effect. For example, treatment of seeds by soaking in Agrimaicin 500 solution (500 g copper sulphate + 30 g oxytetracyclin per kg) in concentrations of 10 g/L of water for 30–60 min eliminated the bacterium from naturally infected seeds. The same treatment of seeds artificially infected with 10^8^ CFU/mL inoculum of the pathogen was, however, ineffective [26]. The use of antibiotics in agriculture is strictly limited in many countries, and the fast evolution of antibiotic-resistant mutants of bacteria necessitates the rotation of antibiotics with other effective means. These are just a few of the reasons why the development of phage control in plant agriculture is considered to be promising. A practical advantage of bacteriophages is their low sensitivity to most pesticides and chemicals [27]. This enables integration of phage treatment with existing plant protection protocols.

The present work estimates the potential of the novel phage Ayka to control legume diseases caused by *Curtobacterium flaccumfaciens* pv. *flaccumfaciens* in planta. The phage demonstrated lytic activity against a number of Cff strains, including those isolated in Russia in 2021. The in vitro infectious properties of phage Ayka are moderate (low progeny and narrow host range), but it can be considered as a prospective component of phage control cocktails.

The results indicate the high *in planta* performance of phage Ayka against infection progress in soybean seedlings after treatment of the seeds inoculated with Cff. Both AUPDC and RGI parameters were higher after phage treatment, compared with conventional treatment with Kocide 2000. Thus, the phage better reduced the growth inhibition caused by Cff infection than copper compounds. A Pre-planting treatment of seeds with inocula of nitrogen-fixing bacteria is an important means of increasing soybean yields [28]. No interaction between phage Ayka, strain *B. japonicum* Semia 5079 and three commercial inoculants permitted for soybean Atuva^®^ Syngenta Rus, Histick^®^ Soy (BASF), and Planteco Soya MC09 (Planteco), was seen in our experiments, which makes it possible to combine these agents as a pre-sowing treatment.

The work also demonstrated a reasonable protective effect of spraying the phage suspension onto above-ground parts of the plant to reduce the Cff population and to prevent the spread of infection. It is necessary, however, to take into account that the experiments were conducted in a glazed greenhouse, whose glass blocked much UV radiation. The fact that ultraviolet light is deadly for phages is one of the major reasons hindering the widespread use of phage control in agriculture. The addition of skimmed milk or other agents to absorb or scatter UV is considered to have prospects for stabilising the phage solution and so promote phage survival [29].

Therefore, the application of phages as the agents of biological control of *C. flaccumfaciens* pv. *flaccumfaciens* can be considered to be a novel ecological strategy for the prevention and cure of bacterial tan spot and wilting in soybeans.

Another problem addressed in this paper is the correct taxonomic attribution of novel phages in accordance with the current ICTV rules. Recently, viral taxonomy has undergone dramatic changes, with the formation of new, higher-ranking taxons [30]. On the one hand, it may seem strange that phages having much in common in terms of their size, morphology and hallmark genomic features, and previously considered to be members of the same genus “φ29-like phages” [31] or subfamily “*Picovirinae*” [32], are now members of different families [33]. On the other hand, this difference reflects the evolutionary distance between the respective bacterial hosts, Bacilli for φ29 and Actynomycetes for Ayka.

The results of intergenomic comparison, gene-sharing network clustering and phylogenetic analysis indicate the need for a thorough taxonomic analysis of phage Ayka. This phage can represent a new taxon related to genera *Anjalivirus*, *Dybvigvirus* and *Badaztecvirus* of unassigned families, and more distantly related to phages belonging to the *Salasmaviridae* and *Guelinviridae* families. The genomic analysis indicated that phage Ayka has a genome organisation similar to the *Anjalivirus*, *Dybvigvirus* and *Badaztecvirus* phages. It is probable that these groups, and phage Ayka, can be re-classified. Interestingly, the phage RHph_N3_8 infecting Gram-negative *Rhizobium* sp. shows substantial closeness to phage Ayka. This might reflect the processes of host switch within the same ecological niche (soil). Another notable observation is the low G+C content of the phage Ayka genome, compared with the genome of *Curtobacterium* strains. This may also be the result of a host switch. Although the genome and replication mechanism of phage Ayka resemble the phages listed above, and according to the results of gene-sharing network analysis the phage belongs to the cluster of so called “φ29-like phages”, the intergenomic distance calculation and the results of phylogenetic analysis indicate a certain degree of distance of the phage from other groups. From the current research, the delineation of new genus *Aykavirus*, based on the VIRIDIC genus threshold of lower than 70%, is proposed. Moreover, based on phylogenetic data, a suggestion is made of further taxonomic refinements, which might include the creation of a new phage subfamily containing the genera of *Anjalivirus*, the novel “*Aykavirus*”, *Badaztecvirus*, and *Dybvigvirus*.

## 4. Materials and Methods

### 4.1. Bacterial Strains and Growth Conditions

Bacterial strains used in this study are listed in Supplementary Table S1. Strains attributed as *C.* *flaccumfaciens* pv. *flaccumfaciens* were isolated from the field in the Stavropol, Ryazan, Orel and Amur regions of the Russian Federation. Their basic characteristics have been reported previously [14]. These strains were complemented with Cff strains from the All-Russian Collection of Microorganisms (VKM; Pushchino, Russia), the French Collection of Phytopathogenic Bacteria (CFBP; Beaucouzé, France), including the type strain CFBP 3418. Initially, the strains were characterized by MALDI biotyper CA System (Bruker, Billerica, MA, USA) for identification. Strain identification was based on four categories using the provided MALDI biotyper database: highly probable species, secure genus, probable species and unreliable identifications according to [15]. Afterwards, strains were attributed by PCR using genus- [16] and species-specific [17] primers. Bacteria were grown at 26 °C in YD broth (YDC media without CaCO_2_) or in Petri dishes with 1.5% agar added. YD with 0.7% agar was used as a top layer for phage experiments.

Strain Semia 5079 of *Bradyrhizobium japonicum* was isolated from a commercial preparation used for soybean seed inoculation and registered as Haikout Super (BASF, Ludwigshafen, Germany) in Russia (Supplementary Table S1). Bacteria were cultured on selective medium BJSM [34]. The isolate was then purified through three rounds of subculturing, and pure isolate used for trials.

### 4.2. Phage Isolation and Purification

Bacteriophage Ayka specific to *C. flaccumfaciens* pv. *flaccumfaciens* was isolated from a soil sample taken from the field in the Penza region. The phage was propagated using Cff strain C089 at 26 °C, according to a previously published protocol [35]. Phage lysate was treated with chloroform and bacterial debris was pelleted by centrifugation at 8000× *g* for 20 min, followed by filtration of the supernatants through 0.22 µm pore-size membrane filters, (Millex-GV, Millipore, Cork, Ireland) and the addition of DNAse I (0.5 mg/mL, 1 h; Evrogen, Moscow, Russia). Phage filtrates were concentrated by ultracentrifugation at 100,000× *g* at 4 °C for 2 h, using a Beckman SW28 rotor (Beckman Coulter, Brea, CA, USA). Phage purification was performed by ultracentrifugation in CsCl step gradient (0.5–1.7 g/mL) at 22,000× *g* for 2 h. Phage-containing opalescent band was collected and dialysed against SM buffer (10 mM Tris-HCl, pH 7.5, 10 mM MgSO_4_, 100 mM NaCl). Phage suspension was stored at 4 °C.

### 4.3. Electron Microscopy

The morphology of phage particles was studied using transmission electron microscopy (TEM). Concentrated purified samples of phage Ayka were placed on grids and stained with 1% aqueous uranyl acetate (pH 4.0). Prepared grids were examined using a JEM-2100 200 kV transmission electron microscope (JEOL, Tokyo, Japan). The dimensions of each phage were averaged among ~20 individually measured particles.

### 4.4. Biological Properties of Phage Ayka

#### 4.4.1. Determination of Phage Host Range

For the host range assessment, phage lytic activity was tested against 74 strains of *Curtobacterium* spp., listed in Supplementary Table S1. The set of strains included 54 entries from the All-Russian Collection of Microorganisms and 20 strains field-isolated in Russia in 2018-2022. For analysis, 5 µL of a phage suspension with a titer of 10^7^ PFU/mL was applied to a double-layer agar containing a bacterial strain, and incubated overnight at 26 °C. The presence of lytic activity was determined by the presence or absence of a sterile plaque in the bacterial lawn at the site of application of the phage suspension. To exclude false positive results, in the case when lysis was observed, it was checked whether the phage forms plaques during titration.

#### 4.4.2. Phage Adsorption and One-Step Growth Experiments

One-step-growth assays were performed according to [36], with some modifications. The host strain C089 was grown in YD broth at 26 °C to OD_600_ ~0.2, then infected with phage Ayka at a multiplicity of infection (MOI) of 0.1. Every 2 min during the early stage of infection, and every 10 min from 20 to 60 min of infection, 100 µL aliquots of culture were taken and transferred to test tubes with 850 µL SM buffer supplied with 50 µL of chloroform. These mixtures were shaken gently for 15 min, to lyse any remaining bacteria. After bacterial lysis, the mixtures were centrifuged and the supernatant was assayed to determine the amount of non-adsorbed or reversibly bound phages by the plaque assay method [37]. The procedure was repeated in triplicate.

One-step growth assays were performed according to [38]. An exponentially growing culture of Cff strain C089 was infected with phage Ayka at MOI of 0.01. Then, the mixture was incubated at 26 °C and aliquots (100 µL) were collected every 20 min, chilled and pelleted by centrifugation (21,000× *g*, 3 min, 4 °C); the supernatants were titered using SM buffer, spread on YD agar plates and incubated overnight at 26 °C. The next day, bacterial colonies were counted. The procedure was repeated in triplicate and the results were averaged. The latent period was determined as the interval between the adsorption of the phages to the bacterial cells and the release of phage progeny. The burst size of phage Ayka was determined as the ratio of the average number of free phage particles after the release phase (plateau average (PFU/mL)) to the respective number during the latency phase (latent average (PFU/mL)).

#### 4.4.3. Phage Stability in Different Conditions

The ability of the phage to survive in different environmental conditions was assessed by incubation of a phage sample (10^6^ PFU/mL in SM buffer) at 4, 10, 20, 30, 40, 50, 60, and 70 °C for 1 h in ThermoMixer F 2.0 (Eppendorf, Hamburg, Germany), addition of a range of buffer solutions (20 mM Tris HCl/20mM Na citrate/20mM Na phosphate) adjusted with NaOH to a pH range 3–12 to a 10^6^ PFU/mL phage Ayka with further incubation at 25 °C for 1 h, and exposure of a phage sample (10^6^ PFU/mL in SM buffer) to UV-B (280–315 nm) radiation using a PL-S9W/12/2p lamp (Philips, Amsterdam, The Netherlands), according to [27]. Treated phages were titrated on YD agar/C089 using the plaque assay method. Procedures were repeated in triplicate and the results were averaged.

#### 4.4.4. Determination of Optimal Multiplicity of Infection (MOI)

Multiplicity of infection was defined as the ratio of virus particles to potential host cells [39], according to the protocol in [40], with modifications. Strain C089 was grown in YD broth at 26 °C to an absorbance OD_600_ of 0.2, measured in a spectrophotometer Nanodrop One (Thermo Fisher Scientific, Waltham, MA, USA). This corresponds to a cell count of approximately 10^8^ CFU/mL. Early log phase cells were infected with phage Ayka at five different ratios (0.001, 0.01, 0.1, 1, 10, and 100 PFU/CFU). After incubation for 6 h at 26 °C, the phage lysate was centrifuged at 21,000× *g* for 3 min at 4 °C. The supernatant was filtered (0.22-µm Millex-GV, Millipore, Cork, Ireland) and the supernatant was titered using YD agar. All assays were performed in triplicate. The MOI resulting in the highest phage titer within 6 h was considered to be an optimal MOI and was used in subsequent large-scale phage production.

### 4.5. Genomic Features of Phage Ayka

#### 4.5.1. Genome Sequencing and Annotation

Bacterial and phage DNA were isolated using the standard phenol-chloroform method after incubation of the sample in 0.5% SDS and 50 µg/mL proteinase K at 65 °C for 20 min. Phage and host bacterium were co-assembled from one sample.

Fragment genome libraries were prepared using 200 ng of genomic DNA as a starting material. DNA was fragmented by ultrasound, using an ME220 focused ultrasonicator (Covaris, Woburn, MA, USA) with the following parameters: iterations—7; duration—10 s; peak power—50; duty factor—20%; cycles per burst—1000. Fragmented DNA was used as an input for library preparation using the NEBNext Ultra II DNA Library Prep Kit for Illumina (New England Biolabs, Ipswich, MA, USA) according to the manufacturer’s instructions. The library was sequenced using an MiSeq sequencer (Illumina, San Diego, CA, USA), using 2 × 250 bp paired-end chemistry, resulting in approximately 435,000 read pairs.

The generated reads were assembled de novo into single contig using SPAdes v. 3.11.1 [41] with default parameters. After assembly, phage-related contigs were extracted by coverage analysis. The genomes were annotated with the assistance of Prokka [42]. The prediction of functions of the encoded phage protein was made through a homology search and an HMM-motif comparison. The homology search was performed by BLAST, using the NCBI non-redundant (nr/nt) database and custom databases made with BLAST using GenBank phage sequences, and the functions were assigned by comparison with known homologues. The presence of tRNA coding regions was checked using tRNAscan-SE [43] and ARAGORN [44]. The average nucleotide identity (ANI) was calculated with orthoANI [45]. Genetic maps were visualised in Geneious Prime 2022. The genome of *Curtobacterium* phage Ayka has been deposited in the NCBI GenBank under Accession number ON381767. The genome of bacterial host Cff C089 (F30-1) was deposited in the NCBI GenBank under Accession number JANUGT000000000.

#### 4.5.2. Intergenomic Comparison and Phylogenetic Analysis

Genomic sequences of reference bacteriophages were downloaded from GenBank. The intergenomic comparison was made with the Virus Intergenomic Distance Calculator (VIRIDIC) [21]. The intergenomic comparison diagrams were made with the Easyfig program [46], using TBLASTX for finding similarities between the encoded proteins.

The alignments were made with MAFFT 7.48, with default settings, using the L-INS-i algorithm [47,48]. The best protein model was found with ModelTest-NG [49]. The phylogenetic trees were constructed with RAxML-NG [50] integrated in raxmlGUI 2.0.7 graphic interface [51], using (ML + transfer bootstrap expectation + consensus) settings, with ten starting trees. The robustness of the RAxML-NG trees was assessed by bootstrapping (1000). The tree was visualised in Geneious Prime 2022.

The gene-sharing network was created using the vConTACT.2.0 pipeline [52], the ProkaryoticViralRefSeq database v94 supplied with the pipeline and the phage genome annotated as described above. The results were visualised using Cytoscape v3.9.0 [53]. The inter-family grouping was produced with the GRAViTy (“Genome Relationships Applied to Virus Taxonomy”) pipeline [54,55], using default settings.

### 4.6. Phage Control of Curtobacterium Infection in Soybean

Experiments on the artificial infection of seeds and plants and phage control were conducted between March and April 2022.

#### 4.6.1. Artificial Infection of Soybean Seeds

The method described in [56], with modifications, was used for the artificial infection of seed rib (hilum). Cff strain C089 was grown for 72 h at 20 ± 2 °C on YD agarised medium. Bacterial suspensions were obtained by dilution in sterile water and concentration adjustment to 1–7 × 10^8^ CFU/mL. The rib of each seed of soybean cv. Kasatka (production year 2021) was pinned with a sterile needle and the seeds were soaked in bacterial suspension (being completely immersed) for 1 h. The mix was then placed in a vacuum at ~10^5^ Pa for 10 min and the seeds were dried on paper towels to remove excessive fluid.

#### 4.6.2. Phage Application on Soybean Seeds

The phage control experiment included four variants: (1) treatment of uninfected seeds with water (negative control); (2) treatment of Cff-infected seeds with water (positive control); (3) treatment of infected seeds with phage (concentration 10^9^ PFU/mL) and (4) treatment of infected seeds with standard bactericide (Kocide^TM^ 2000, WDG (copper hydroxide 350 g/kg) Corteva Agriscience Russia, Rostov-on-Don, Russia) solution at 10 g/L, according to [26].

Phage treatment of infected seeds was processed as follows. Aliquots of 25 g seeds (average mass for cv. Kasatka is ~122.4 g per 1000 pieces of seeds) were weighed in 50 mL Falcon tubes, phage solution was added, and the tube was vortexed for 1–2 min for sorption of the solution on the seeds, thus emulating an industrial seed pickler. Treated seeds were planted in the turf/perlite substrate (Veltorf, Velikie Luki, Russia) in 40-cell plastic transplant trays (cell volume 0.12 L, AgrofloraPack, Vologda, Russia). The plants were grown in the greenhouse at 28/22 °C (14-h day/10-h night) with natural insolation and watered as needed. The treatments in each experiment were arranged according to a complete randomisation design. There were three replicates in each treatment with 40 seeds (one tray per replicate).

On days 15, 18, 21, 24, 27 and 31 after seeding, the development of bacterial wilt for each plant was estimated according to [56] in a 0-5 range, where 0 = no wilting symptoms; 1 = wilting of one of the primary leaves; 2 = wilting of both primary leaves but not of the first ternary leaf; 3 = wilting of first ternary leaf; 4 = seedling death after development of primary leaves; 5 = no seedlings or complete wilting of plants. AUPDC (Area Under Progress Disease Curve) was calculated according to [57], using the same scale, in MS Excel 2007. The phenotypic assessment scale was adapted for soybean and is presented in Supplementary Figure S11.

Simultaneously with the assessment of disease symptoms, the height of each plant was measured from leaf petiole to apical bud. The Relative Growth Index (RGI) was calculated as RGI = (LnP2 − LnP1)/(T2 − T1), where Ln = natural logarithm, P2 and P1 = plant height at moments T2 (endpoint) и T1 (starting point), according to [58].

#### 4.6.3. Artificial Infection of Soybean Leaves

Cff infection of vegetating soybean cv. Kasatka plants was achieved by bacterial infiltration into the soybean leaf using a 1113 AirControl airbrush (JAS, Ningbo, China), according to [59], with modifications. A bacterial suspension was prepared as for seed infection, with the addition of wetting agent Silwet Gold (Chemtura, Philadelphia, PA, USA) to a concentration 0.01% (*v*/*v*). Ternary leaves of plants were infiltrated on a V2 stage pressing the leaf to a flat surface (Petri dish) to avoid pressure damage from the airbrush spray. All leaves were infiltrated with an average dose of 5 mL suspension, with a concentration of 10^9^ CFU/mL per plant. The negative control was sprayed with an equivalent amount of water, using a wetting agent. The plants were grown in 0.5 L plastic pots on a turf/perlite substrate, in a greenhouse, at an average temperature day/night of 28/22 °C. Watering was carried out daily by sprinkling. Two days before, and 24 h after inoculation, relative humidity was maintained at ~95%.

#### 4.6.4. Phage Treatment of Soybean Plants

Experiments on phage control of leaf infection included four variants: (1) treatment of Cff-infected leaves with water (positive control); (2) application of phage suspension (adjusted to 10^9^ PFU/mL with sterile water) 1 h after infection; (3) simultaneous inoculation of leaves with phage (10^9^ PFU/mL) and bacteria (10^9^ CFU/mL), mixed immediately before application; (4) treatment with standard bactericide Kocide^TM^ 2000, WDG (10 g/L). In all cases, ~5 mL of working solution was applied to each plant with an airbrush (droplet size ~300 µm), to complete wetting of the leaves. Further cultivation of plants proceeded as mentioned previously.

An assessment of the development of the disease, in terms of the infection of adult plants, was carried out on the 12th day after infection (Figure 6), using the LeafDoctor application (https://www.quantitative-plant.org/software/leaf-doctor, accessed on 21 October 2021) installed on an iPhone SE 2. All leaves from all plants were individually photographed and analysed by moving the threshold slider until only symptomatic tissues were transformed into a blue shade, and calculating the percentage of diseased tissue according to the developer’s recommendations [60].

Statistical processing of the analysed data was carried out using the method of variance analysis, with Statistica 12.0 (StatSoft, TIBCO, Palo Alto, CA, USA), comparing averages according to the Duncan criterion. Percentage data were converted to arcsines before processing. Plots were created with GraphPad Prism 9.2.0 (GraphPad Software Inc., Los Angeles, CA, USA).

## Data Availability

The annotated genomic sequence of *Curtobacterium* phage Ayka has been deposited to NCBI GenBank and is available under accession number ON381767. The genome of the bacterial host Cff C089 (F30-1) has been deposited to NCBI GenBank and is available under accession number JANUGT000000000 (BioProject PRJNA863744).

## Supplementary Materials

The supporting information can be downloaded at: https://www.mdpi.com/article/10.3390/ijms231810913/s1.

## Abbreviations

cv	cultivar
WDG	Water-Dispersible Granules
CFU	colony-forming unit
PFU	plaque-forming unit
