# Supplementary material for: Ayka, a Novel Curtobacterium Bacteriophage, Provides Protection against Soybean Bacterial Wilt and Tan Spot
Source: Int J Mol Sci. 2022 Sep 18;23(18):10913. doi: 10.3390/ijms231810913 (PMC9502298; doi:10.3390/ijms231810913)
Supplement: Supplementary file 1 [file ijms-23-10913-s001.zip › supplementary figures (summary).pdf]

International Journal of *Molecular Sciences*

Ayka, a Novel *Curtobacterium* Bacteriophage, Provides Protection Against Soybean Bacterial Wilt and Tan Spot

Rashit I. Tarakanov, Anna A. Lukianova, Peter V. Evseev, Roxana I. Pilik, Anna D. Tokmakova, Eugene E. Kulikov, Stepan V. Toshchakov, Alexander N. Ignatov, Fevzi S.-U. Dzhalilov, and Konstantin A. Miroshnikov

Supplementary materials.

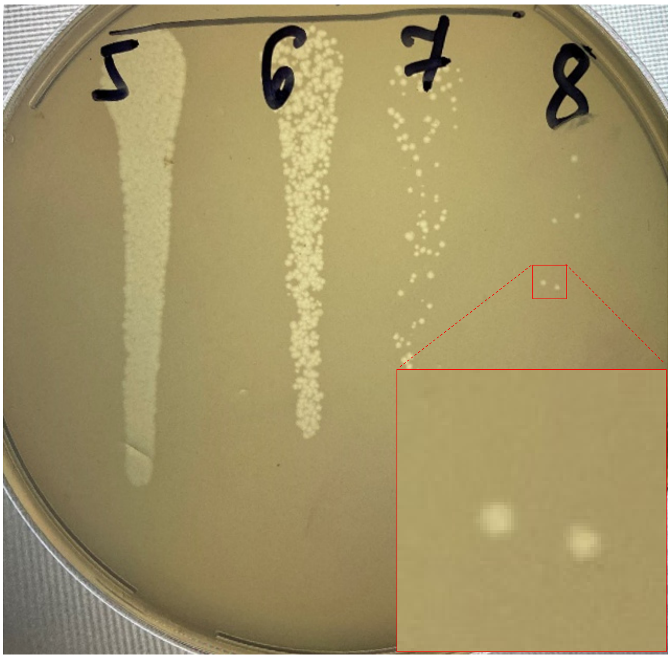

Supplementary Figure S1. Ayka phage plaques shape on 0.7% upper agar YD with the host bacterium strain C089. The numbers 5, 6, 7 and 8 on the plate indicate the numbers of tenfold dilutions of the phage suspension in the SM buffer. In each dilution, 10 µl of suspension was dripping and the titer was determined by the drip drop method after 24 h cultivation pn 28°C.

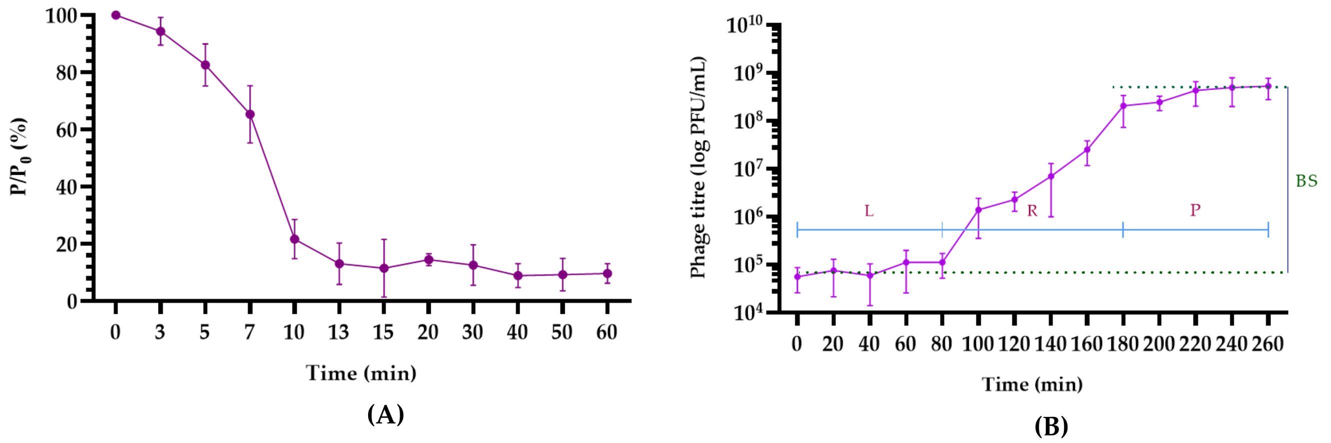

Supplementary Figure S2. Phage adsorption curve (A) and single-stage phage growth curve (B) of bacteriophage Ayka. *C. flaccumfaciens* pv. *flaccumfaciens* C089 was used as a host. The ordinate axis shows the ratio of the current title at each time (P) to the original (Po) multiplied by 100%. L - latent phase; R - virion release phase; P - plateau phase; BS - burst size. Values in panels represent the mean of three independent trials, respectively, and error bars represent the standard deviation.

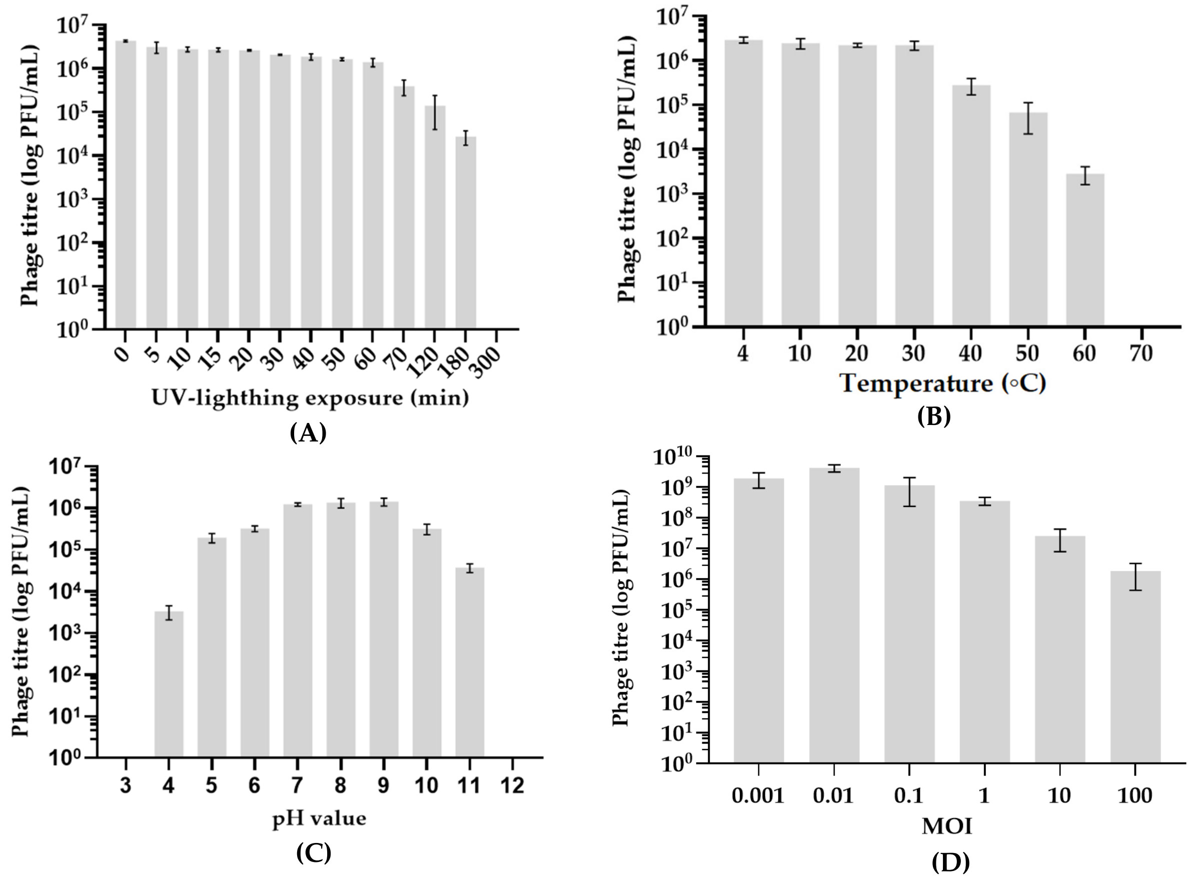

Supplementary Figure S3. Survival of *Curtobacterium flaccumfaciens* pv. *flaccumfaciens* bacteriophage Ayka under different stress factors and optimal multiplicity of infection. The phages were treated by UV irradiation from 5 to 300 min (A), by temperatures from 4 to 70°C for 1 h (B), and by pH from 3 to 12 for 1 h (C). Comparison of phage titer after

incubation for 6 h at five ratios of MOI (0.001, 0.01, 0.1, 1, 10 and 100 PFU/CFU) in YD medium (D). All tests were repeated 3 times. Standard deviation (sd) is shown for each bar.

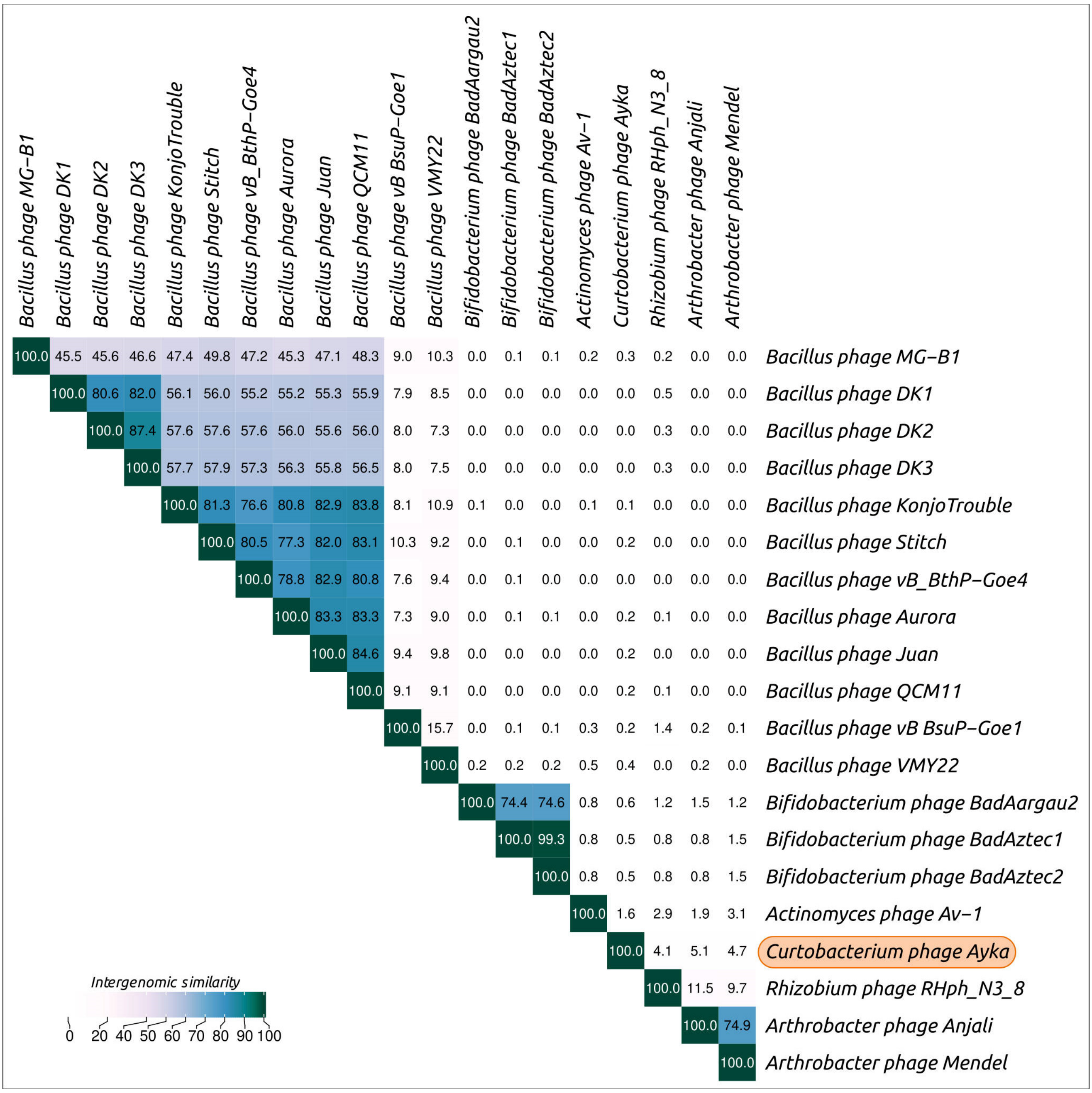

Supplementary Figure S4. VIRIDIC generated heatmap of *Curtobacterium* phage Ayka and related phages. The colour coding indicates the clustering of the phage genomes based on intergenomic similarity. The numbers represent the similarity values for each genome pair, rounded to the first decimal.

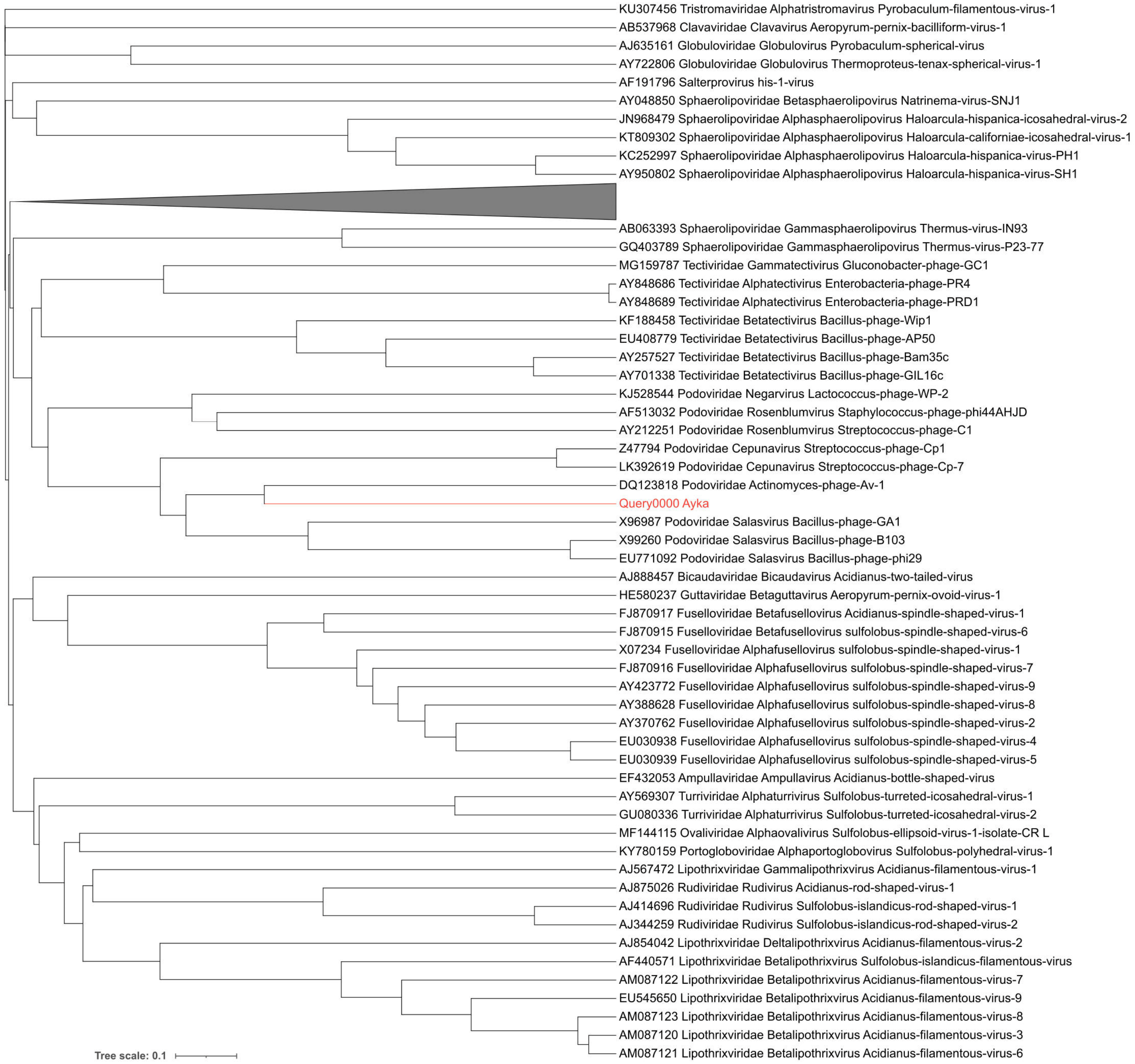

Supplementary Figure S5. Phylogenetic trees constructed with GRAViTy using genomic sequence of phage Ayka and GRAViTy default database.

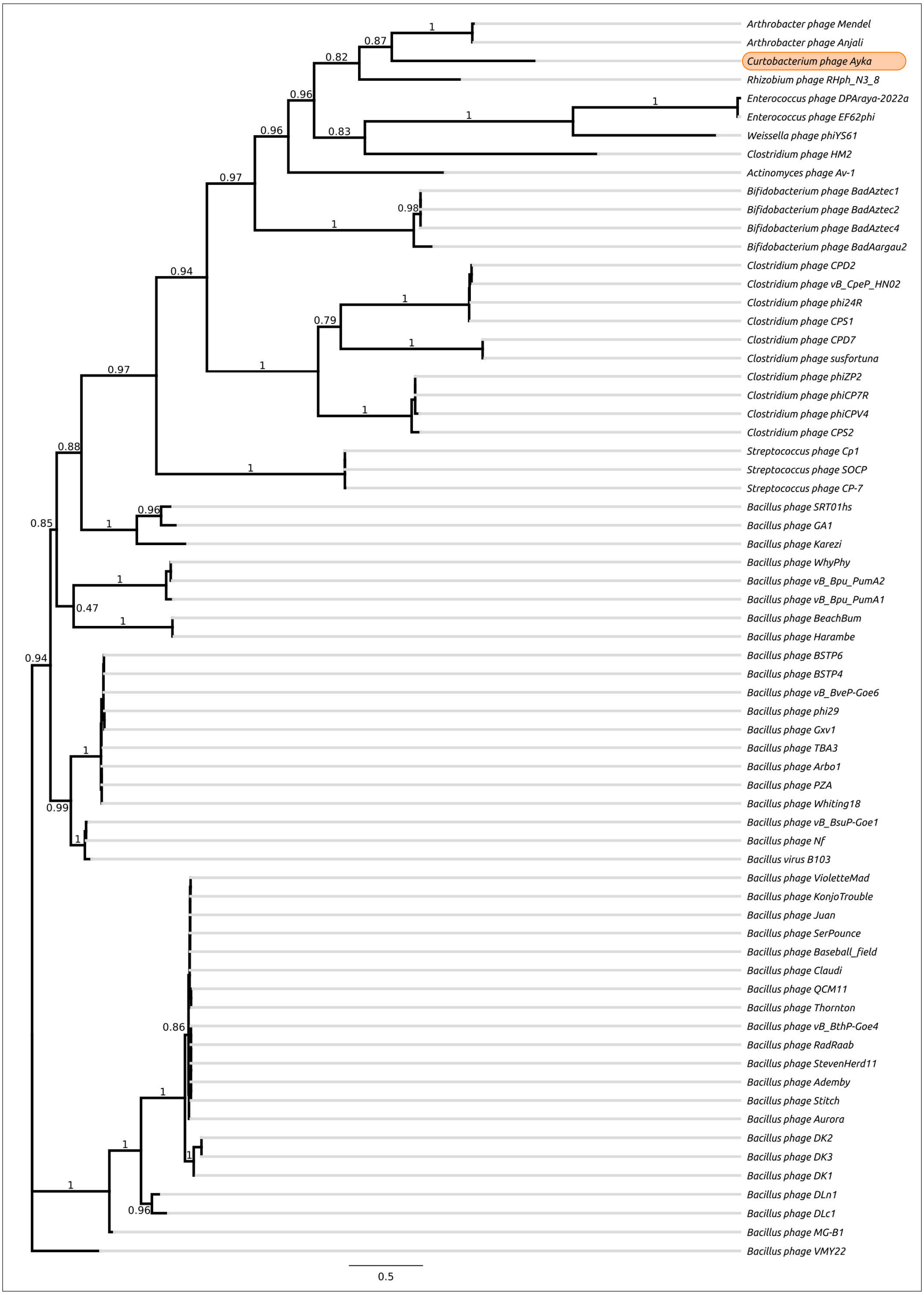

Supplementary Figure S6. Best-scoring phylogenetic trees constructed with RAxML-NG based on the protein sequences of connector protein. Bootstrap support values are shown above their branch as a percentage of 1000 replicates. The scale bar shows 0.5 estimated substitutions per site and the tree was unrooted.

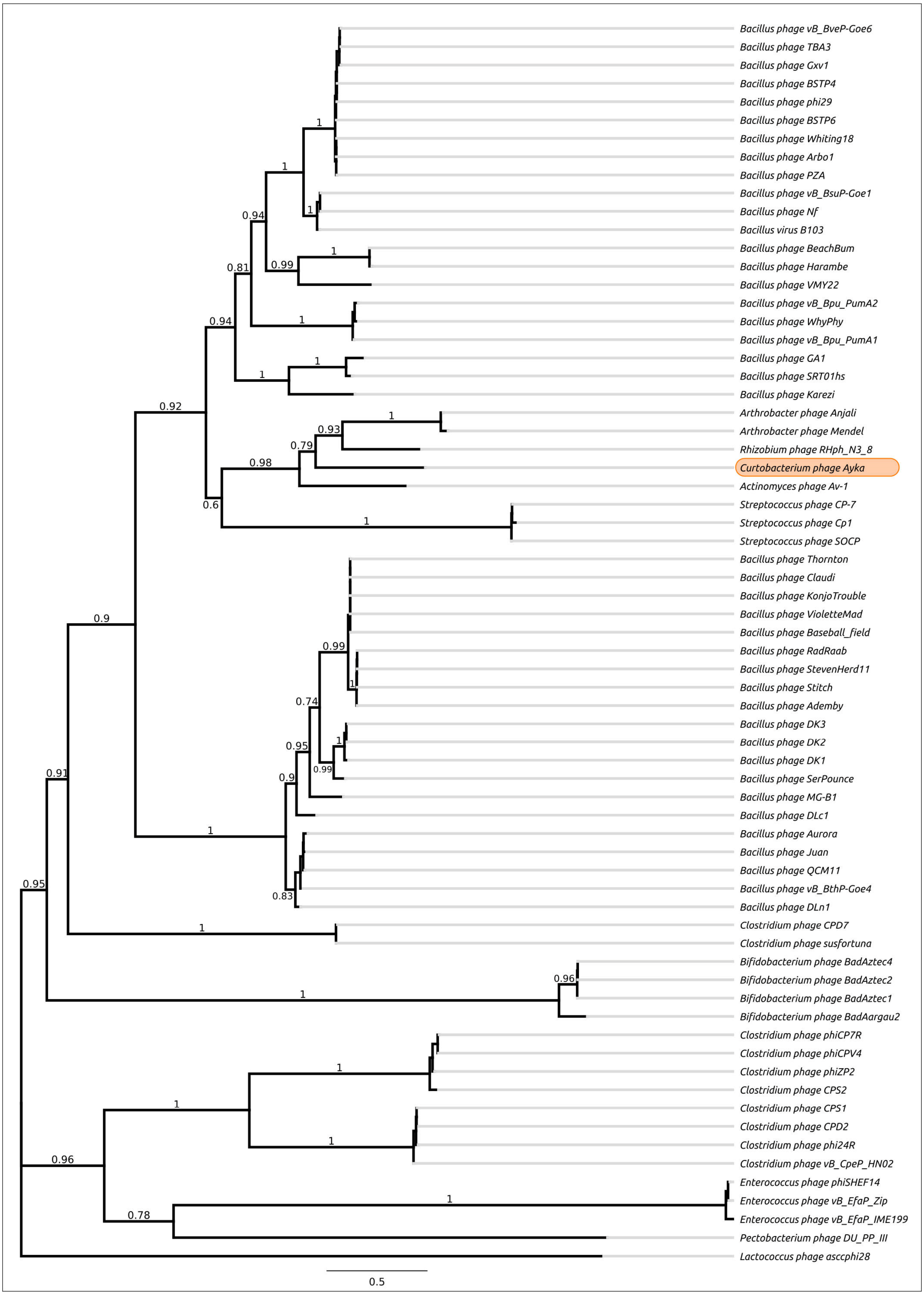

Supplementary Figure S7. Best-scoring phylogenetic trees constructed with RAxML-NG based on the protein sequences of DNA polymerase. Bootstrap support values are shown above their branch as a percentage of 1000 replicates. The scale bar shows 0.5 estimated substitutions per site and the tree was unrooted.

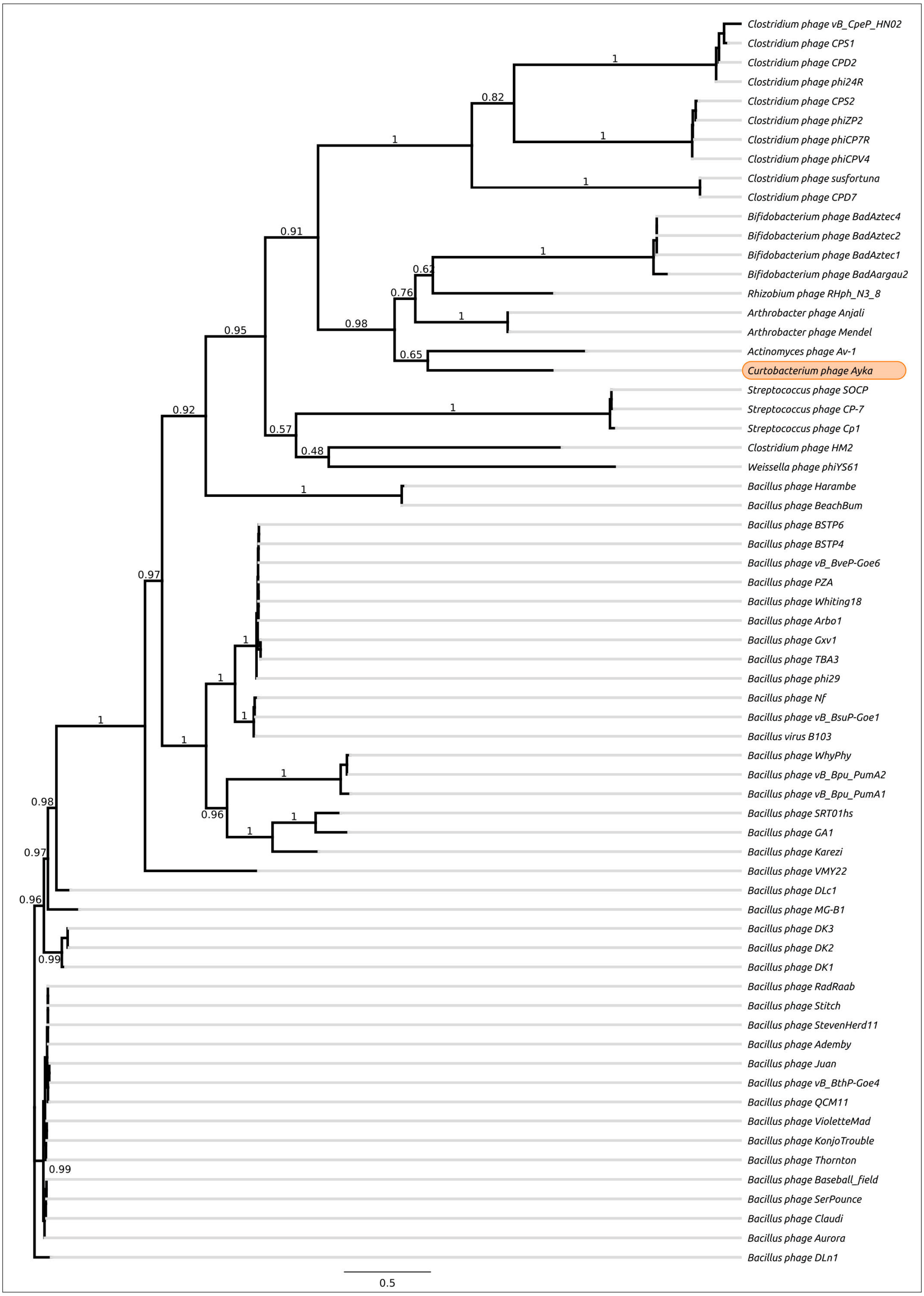

Supplementary Figure S8. Best-scoring phylogenetic trees constructed with RAxML-NG based on the protein sequences of major capsid protein. Bootstrap support values are shown above their branch as a percentage of 1000 replicates. The scale bar shows 0.5 estimated substitutions per site and the tree was unrooted.

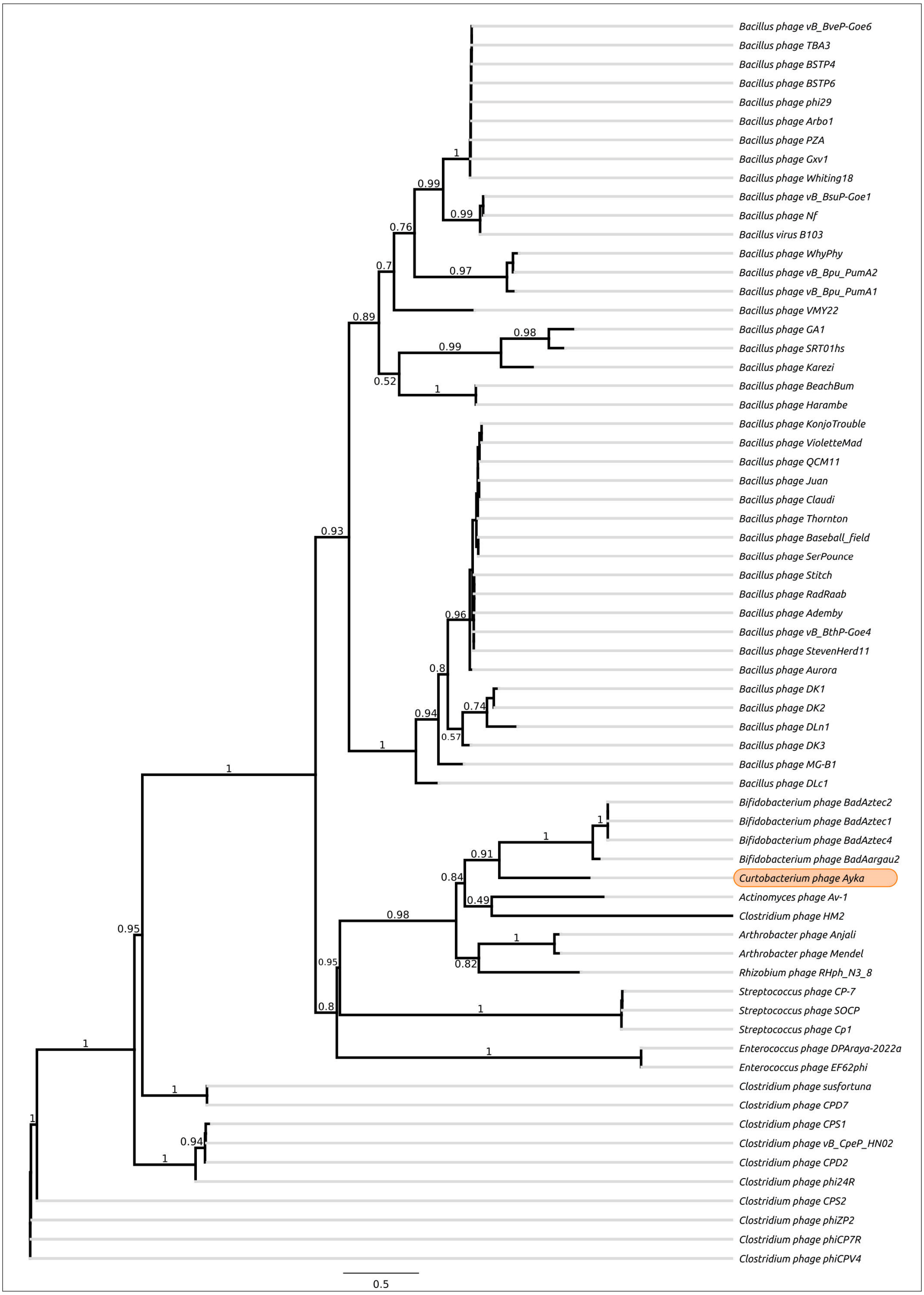

Supplementary Figure S9. Best-scoring phylogenetic trees constructed with RAxML-NG based on the protein sequences of proximal tail tube connector protein. Bootstrap support values are shown above their branch as a percentage of 1000 replicates. The scale bar shows 0.5 estimated substitutions per site and the tree was unrooted.

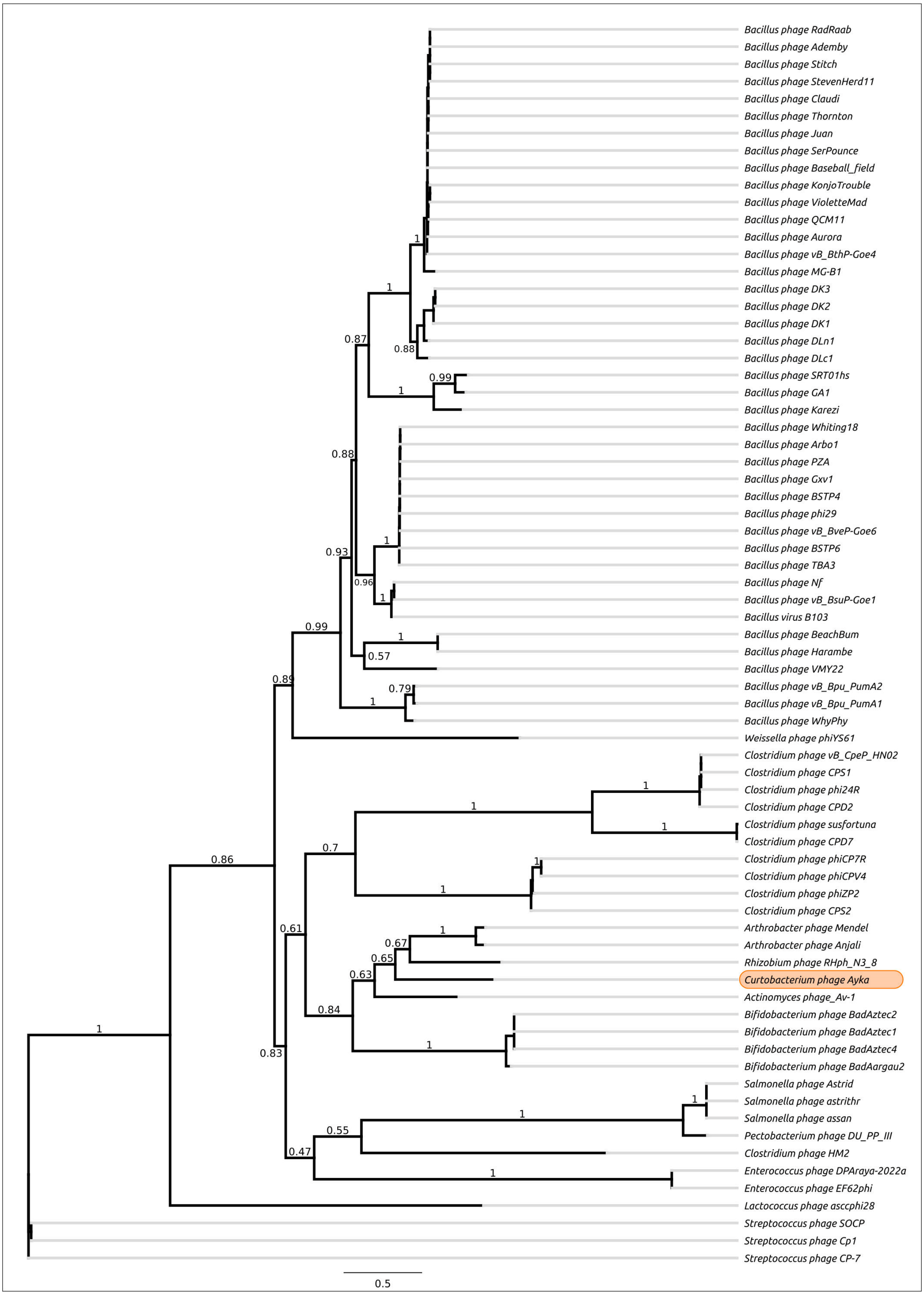

Supplementary Figure S10. Best-scoring phylogenetic trees constructed with RAxML-NG based on the protein sequences of terminase. Bootstrap support values are shown above their branch as a percentage of 1000 replicates. The scale bar shows 0.5 estimated substitutions per site and the tree was unrooted.

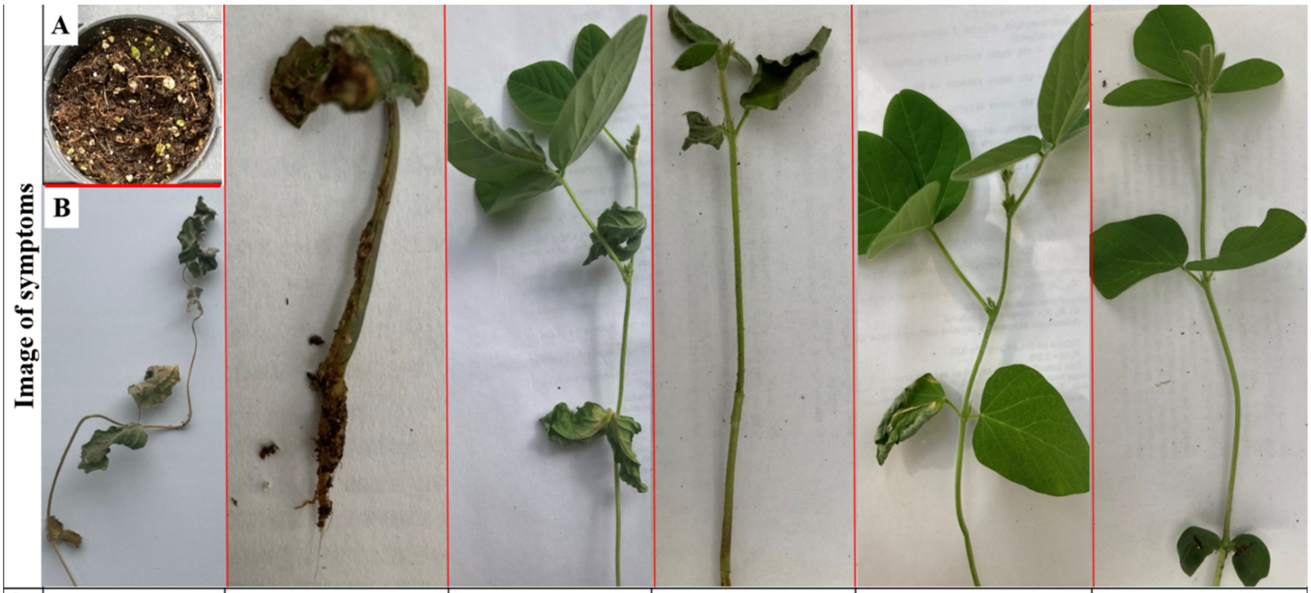

Sco re	5	4	3	2	1	0
Description	no seedlings (A)/ complete withering and loss of turgor (in adult plants) (B)	the death of a seedling after the development of the first leaves	Wilting of the first ternary leaf	wilting on both primary leaves, but not on the first ternary leaf	wilting on one of the primary leaves	no symptoms of wilting

Supplementary Figure S11. Rating scale for evaluation of wilting during infection of soybean seeds Cff, adapted for soybean from Ref [56]. A score of 5 indicates the death of the plant before seedlings (**A**) or the complete wilting of the adult plant during accounting (**B**).
